# Spinal cord injury induces astroglial conversion towards neuronal lineage

**DOI:** 10.1186/s13024-016-0133-0

**Published:** 2016-10-06

**Authors:** Harun Najib Noristani, Jean Charles Sabourin, Hassan Boukhaddaoui, Emilie Chan-Seng, Yannick Nicolas Gerber, Florence Evelyne Perrin

**Affiliations:** 1 University of Montpellier, Montpellier, F-34095 France; 2 INSERM U1198, Place Eugène Bataillon CC105, 34095, Montpellier, Cedex 5 France; 3 EPHE, Paris, F-75014 France; 4 INSERM U1051, F-34095 Montpellier, France; 5 Integrative Biology of Neurodegeneration”, IKERBASQUE Basque Foundation for Science and Neuroscience Department, University of the Basque Country, E-48013 Bilbao, Spain; 6 Department of Neurosurgery, Gui de Chauliac Hospital, F-34295 Montpellier, France

**Keywords:** Spinal cord injury, Astrocytes, Astrogliosis, Transdifferentiation, Cell specific transcriptomic

## Abstract

**Background:**

Neurons have intrinsic capability to regenerate after lesion, though not spontaneously. Spinal cord injury (SCI) causes permanent neurological impairments partly due to formation of a glial scar that is composed of astrocytes and microglia. Astrocytes play both beneficial and detrimental roles on axonal re-growth, however, their precise role after SCI is currently under debate.

**Methods:**

We analyzed molecular changes in astrocytes at multiple stages after two SCI severities using cell-specific transcriptomic analyses.

**Results:**

We demonstrate that astrocyte response after injury depends on both time after injury and lesion severity. We then establish that injury induces an autologous astroglial transdifferentiation where over 10 % of astrocytes express classical neuronal progenitor markers including βIII-tubulin and doublecortin with typical immature neuronal morphology. Lineage tracing confirmed that the origin of these astrocytes is resident mature, rather than newly formed astrocytes. Astrocyte-derived neuronal progenitors subsequently express GABAergic, but not glutamatergic-specific markers. Furthermore, we have identified the neural stem cell marker fibroblast growth factor receptor 4 (Fgfr4) as a potential autologous modulator of astrocytic transdifferentiation following SCI. Finally, we establish that astroglial transdifferentiation into neuronal progenitors starts as early as 72 h and continues to a lower degrees up to 6 weeks post-lesion.

**Conclusion:**

We thus demonstrate for the first time autologous injury-induced astroglial conversion towards neuronal lineage that may represent a therapeutic strategy to replace neuronal loss and improve functional outcomes after central nervous system injury.

**Electronic supplementary material:**

The online version of this article (doi:10.1186/s13024-016-0133-0) contains supplementary material, which is available to authorized users.

## Background

Clinical symptoms linked to spinal cord injury (SCI) depend on the anatomical level and lesion severity, ranging from minor sensory/motor impairment to complete tetraplegia. Following SCI, a glial scar surrounds the lesion site constituting a real physicochemical barrier that is a major obstacle to axonal re-growth [1]. Glial scar is composed of two cell types, namely microglia and astrocytes. Although increased astrocyte reactivity (also referred as astrogliosis) is a prominent pathological hallmark after CNS lesion, its precise role after SCI has been under debate for a long time [2]. Activated astrocytes over-express transcription factors that upsurges inflammation and inhibits axonal sprouting associated with worsening of functional recovery after SCI [3]. Concomitantly, astrocytes also play protective roles after injury via secretion of anti-inflammatory factors [4] and isolation of un-damaged tissue [5].

These studies highlight the beneficial and detrimental roles of astrocytes after SCI raising the question whether their contradictory responses may be due to differences in injury severity, time after injury or both. Detailed molecular analysis of pure astrocyte at acute and chronic stages after different lesion severities may accurately uncover their precise contribution in SCI pathophysiology. Indeed, recent studies, including our own, using cell-specific transcriptomic analyses have been critical to better understand the role of glial cells in numerous CNS pathophysiology [6–14].

Here, we used Aldh1l1-EGFP transgenic mice that express enhanced green fluorescent protein (eGFP) in astrocytes. Using fluorescence-activated cell sorting (FACS), we isolated pure astrocyte population and carried out RNA sequencing (RNA-Seq) to study the molecular signature of astrocytes after hemisection (HS) and full transection (FT) compared to that of non-injured (NI) controls. We have chosen FT since no regeneration can ever occur through the lesion site and lateral HS because limited spontaneous regeneration does occur due to the presence of undamaged tissue. We identified pronounced transcriptional deregulations in astrocytes following SCI that are driven by time post-injury and lesion severity. We also identified injury-induced astroglial transdifferentiation towards neuronal lineage where resident mature astrocytes over-expressed the neural stem cell marker fibroblast growth factor receptor 4 (Fgfr4), the neuronal progenitor markers βIII-tubulin and doublecortin (DCX). In addition, these astrocytes also transformed into classical neuronal progenitor-like morphology with bi-polar processes. Subsequently, astrocyte-derived neuronal progenitor cells expressed markers typically associated with mature GABAergic interneurons. More importantly, we demonstrate that astroglial transdifferentiation into neuronal progenitor starts as early as 72 h post-lesion and continues up to 6 weeks after both HS and FT. These data demonstrate a novel insight into glial cell plasticity after injury and provide therapeutic targets where autologous astroglial transdifferentiation could be enhanced to replace lost neurons and improve functional outcomes after SCI.

## Methods

### Experimental procedures

Transgenic mice expressing eGFP in astrocytes ((Aldh1l1-EGFP)OFC789Gsat/Mmucd) were purchased from the Mutant Mouse Regional Resource Centre (MMRRC) and maintained on a Swiss Webster strain background. Aldehyde dehydrogenase 1 family member L1 is a specific pan-astrocytic marker [15]. Mice were housed in controlled conditions (hygrometry, temperature and 12 h light/dark cycle).

#### Spinal cord injury

Adult mice (12 weeks) were anesthetized by inhalation of isoflurane gas (1.5 %); laminectomy was performed and either a lateral hemisection (HS) or full transection (FT) was carried out under a microscope using a micro-scalpel (FST, Heidelberg, Germany), as described previously [16]. Lesions were done at thoracic 9 level to obtain hemi paraplegia (HS) or complete paraplegia (FT) while preserving full respiratory function.

#### Postoperative cares

Bladder was emptied manually twice daily until recovery of full sphincter control (for HS) or throughout the study period (for FT). Bodyweight was measured before surgery and daily throughout the 6 weeks period after injury.

#### FACS

Fluorescence Assisted Cell Sorting (FACS) was used to isolate pure populations of astrocytes from Aldh1l1-EGFP transgenic mice. We used a 1 cm-segment centered on the lesion site to isolate eGFP^+^ astrocytes. For non-injured controls, the equivalent 1 cm-segment of the thoracic spinal cord was used. Male Aldh1l1-EGFP mice were anesthetized with tribromoethanol (500 mg/kg) and intracardially perfused with RNAse-free 0.1 M phosphate base saline (PBS, Invitrogen, Carlsbad, USA). Spinal cords were dissected and dissociated in 750 μl PBS, 100 μl trypsin 13 mg/ml, 100 μl hyaluronidase 7 mg/ml, 50 μl kinurenic acid 4 mg/ml (all from Sigma Aldrich, Saint Louis, USA) and 20 μl DNAseI 10 mg/ml (Roche, Rotkreuz, Switzerland) for 30 min at 37 °C. Gentle mechanic dissociation was carried out by pipetting. Cell suspension was sieved on a 40 μm sieve (BD Biosciences, Franklin Lakes, USA), re-suspended in PBS-0.9 M sucrose and centrifuged for 20 min at 750 g. The pellet was re-suspended in 500 μl of 7-aminoactinomycin D (7-AAD) 1 μl/ml. Living astrocytes were sorted using FACS ARIA (BD Biosciences, Franklin Lakes, USA), equipped with a 488 nm Laser Sapphire 488–20. Size threshold was used to eliminate cellular debris. Isolated astrocytes were then centrifuged for 5 min at 700 g and re-suspended in 250 μl of RLT lysis buffer (Qiagen, Maryland, USA) and 1 % beta-mercaptoethanol. For flow cytometry analysis of eGFP and βIII-tubulin co-expression, after sieving and sucrose, cells were incubated for 20 min on ice in mouse anti βIII-tubulin in PBS (1:100; MAB1195; R&D Systems, Minneapolis, USA). Cells were then centrifuged for 5 min at 400 g, washed with cold PBS and incubated for 15 min on ice in Allophycocyanin (APC)-conjugated donkey anti mouse in PBS (1:100; 715-136-150; Jackson Immunoresearch, Carlsbad, USA). Controls were done using wild type mice (to show cell distribution without any staining), non-lesion segment alone (to show eGFP expression alone), wild type stained with βIII-tubulin (to show APC staining alone) as well as without primary antibody. Cells were centrifuged for 5 min at 400 g, washed with cold PBS and re-suspended in 7-AAD before sorting.

#### RNA sequencing

Total RNA was isolated using RNeasy Mini Kit, (Qiagen, Maryland, USA) including DNAse treatment. The quality of starting RNA and amplified cRNA were tested (Agilent 2100 bioanalyzer, RNA 6000 Pico LabChip, Palo Alto, USA) and proceeded only if the RNA integrity number (RIN) was > 7. Astrocytic RNA was isolated at 1 and 2 weeks after injury (*n* = 18 for HS 1 week and *n* = 16 for HS 2 weeks; *n* = 6 for FT 1 week and *n* = 34 for FT 2 weeks) as well as NI controls (*n* = 13). RNA-Seq was performed on the polyadenylated fraction of RNA. Three biological replicates were used for each of the mentioned time-points including NI control in all experimental conditions. 10 ng of total RNAs were used for each sequencing library. Sequencing quality control was done using FastQC. The reads containing adapter sequences were removed with FASTX-Toolkit. The filtered reads were mapped with TopHat v2.0.9 software in order to use the UCSC mm10 release reference on new junctions and known annotations. Biological quality control and summarization were done using RSeQC v2.3.3, PicardTools v1.92 and SamTools v.1.18. The count data were prepared with HTSeq v.0.5.4p3. Data normalization and differential expression analysis was performed using R/Bioconductor edgeR software v.3.3.6. Very lowly expressed genes were filtered out whilst genes that achieved 10 counts in at least one condition were kept. The filtered data were normalized by the library size and differentially expressed genes were estimated using negative binomial general model statistics. To identify transcripts that were differentially expressed, we defined a criterion of a 2-fold and greater difference plus a significant (*p* < 0.05) false discovery rate (FDR). Statistics: *t*-test with un-equal variance. The R/Bioconductor MFuzz package v.2.18.0 was used for fuzzy clustering of the differentially expressed genes over time in both HS and FT compared to NI controls. Pathway analysis was done using MetaCore, as previously described [10].

#### Primary antibodies for immunological staining

Primary antibodies used included rabbit anti GFAP (1:1000; Z 0334; Dako, Glostrup, Denmark), mouse anti GFAP (1:1000; G3893; Sigma Aldrich, Saint Louis, USA), mouse anti MAP2 (1:500; M-1406; Sigma Aldrich, Saint Louis, USA), mouse anti-MBP (1:500; MAB 384; Millipore, California, USA), rabbit anti Iba1 (1:1000; 019–19741; Wako Pure Chemical Industries, Osaka, Japan), chicken anti GFP (1:1000; Ab13970; Abcam, Cambridge, UK), mouse anti βIII-tubulin (1:500; MAB1195; R&D Systems, Minneapolis, USA), goat anti Fgfr4 (1:500; AF2265; R&D, Minneapolis, USA), rabbit anti GAD 65/67 (1:500; AB1511; Millipore, California, USA), guinea pig anti Tlx3/Rnx (1:500; kindly provided by Thomas Müller and Carmen Birchmeier, Max-Delbrück-Center, Berlin, Germany), mouse anti NeuN (1:500; MAB377; Millipore, California, USA), rabbit anti doublecortin (1:500; ab18723; Abcam; Cambridge, UK), rat anti BrdU (1:500; Ab6326; Abcam; Cambridge, UK) and mouse anti parvalbumine (1:500; P3088, Sigma Aldrich, Saint Louis, USA).

#### Immunohistochemistry

Hemisected and non-injured control female mice were anesthetized with tribromoethanol (500 mg/kg, i.p) and perfused intracardially with 0.1 M PBS followed by 4 % paraformaldehyde (PFA, Sigma Aldrich, Saint Louis, USA). Spinal cords were removed and post-fixed for 2 h in 4 % PFA, cryoprotected in sucrose 30 %, included in Tissue Tek (Sakura, Alphen aan den Rijn, The Netherlands), frozen and kept at −20 °C until processing. Frozen spinal cords were cut either longitudinally (20 μm) or transversally (14 μm). All sections were collected on super frost plus slides.

For fluorescence immunohistochemistry, slides were washed in PBS, treated for 20 min in 0.1 M PBS containing lysine (20 mM, pH 7.2). Sections were then blocked for 1 h with 0.1 M PBS containing bovine serum albumin (BSA, 1 %, Sigma Aldrich, Saint Louis, USA) and triton X-100 (0.1 %, Fisher Scientific, Illkirch, France) followed by incubation in primary antibody for 48 h at 4 °C. Slides were then washed in 0.1 M PBS and were placed in corresponding secondary antibodies conjugated to Alexa 488, 594 or 633 (Vector Laboratories, Burlingame, USA and Millipore Bioscience Research Re-agents, Massachusetts, USA). The nuclear stain, 4′,6-diamidino-2-phenylindole dihydro-chloride (DAPI, 2 ng/ml, Invitrogen, Massachusetts, USA) was used as a general nucleus marker. Sections were cover slipped using fluorescent mounting medium (DAKO, Denmark). For BrdU detection, sections were pre-incubated in 2 N HCl for 30 min of DNA denaturation before fluorescence immunohistochemistry.

Morphometric fluorescent photographs were obtained using laser scanning inverted (Leica SP5, Mannheim, Germany) and (Zeiss 5 Live Duo, Oberkochen, Germany) confocal microscopy to unveil the co-expression of two proteins. 225 × 225 μm^2^ images were taken adjacent to the lesion center and the colocalization coefficient were measured between the two channels. 4–7 animals were used at each given time-point. Colocalization analysis was performed using the Carl Zeiss LSM-710-NLO software.

#### Area fraction

To determine Fgfr4 protein expression, the area fraction was measured in at least 1000 μm^2^ surface area rostral and 1000 μm^2^ caudal to the lesion sites at multiple time-points after SCI. Area fraction is a sensitive and reliable method to measure expression level of a given signal and to detect changes caused by experimental manipulations. To avoid potential variation in staining intensity between different slides or animals, we had carried out immunostaining of all animals in parallel. Immunofluorescent-labelled spinal cord sections were imaged using constant exposure time in randomly selected longitudinal section with clearly visible lesion site (Zeiss, Oberkochen, Germany). Area fraction quantification was performed using ImageJ software (NIH, USA). Threshold was adjusted according to non-injured control and was kept constant for all images. Area fraction was analyzed individually both rostral and caudal to the lesion site. For non-injured controls, we analyzed Fgfr4 expression in equivalent surface area within the thoracic spinal cord segment. Five time-points after injury were analyzed for Fgfr4 expression level namely, 72 h, 1, 2, 4 and 6 weeks after lesion with 5 animals per groups.

#### BrdU injection after SCI

BrdU (Sigma Aldrich, Saint Louis, USA), was administered by intraperitoneal injections at 100 mg/kg dissolved in saline. Each animal was given a single daily injection immediately after lesion through 7 days after SCI.

#### Statistical analysis

One-way analysis of variance was used to compare differences over-time. Un-paired t-tests were used to compare differences between two independent groups. Significance was accepted at p ≤ 0.05. The data were analyzed using GraphPad Prism 4.0 (GraphPad Software, Inc, CA, USA). All data are shown as the mean ± standard error of the mean (SEM).

## Results

### Time and severity-dependent astrocytic response after SCI

Dual immunohistochemistry in Aldh1l1-EGFP mice using classical astroglial marker glial fibrillary acidic protein confirmed specific eGFP expression in astrocytes (Additional file 1: Figure S1a–c). No eGFP expression was observed in microglia/macrophages, oligodendrocytes and neurons (Additional file 1: Figure S1d–g). Using FACS we isolated pure astroglial populations with high RNA quality (Additional file 1: Figure S1h–k). We then carried out RNA-Seq analysis to uncover molecular changes in astrocytes at multiple stages after two SCI severities. Analysis of deregulated transcripts showed specific up-regulation of classical astroglia-specific marker genes (*gfap* and *serpina3n*) and no dysregulation in neuronal (*nefl*, *Syt1*, *Gabra1*, and *Snap25*) and oligodendrocytes (*Mog*, *Sox10*, *Cx47* and *Mbp*) transcripts [14] that confirms sample purity (Additional file 2: Table S1).

**Fig. 1:**
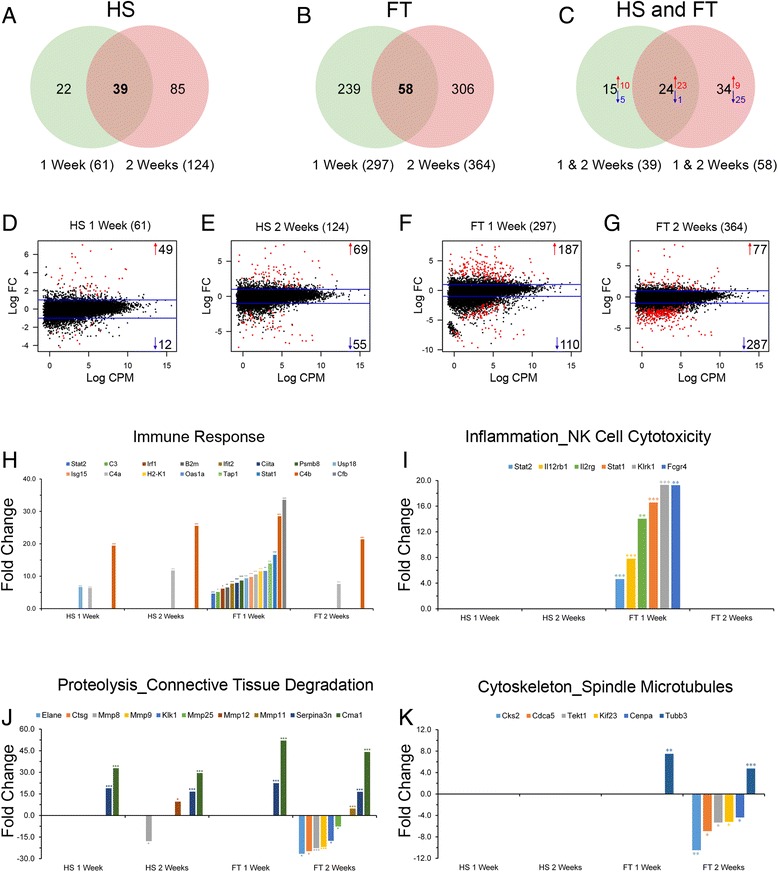
Astrocytic transcriptomic alterations after SCI. Venn diagrams indicating the number of deregulated genes in FACSed astrocytes (**a**–**c**) at different stages after HS and FT. Relationship between average gene expression and log fold-change in astrocytes (**d**–**g**) at different stages after HS and FT injuries. Bar graphs displaying astrocytic involvement in different pathways (**h**–**k**) at multiple time-points after HS and FT SCI. At 1 week after FT, astrocytes predominantly over-express genes that are involved in immune response and inflammation (**h** & **i**). At 2 weeks after FT, astrocytes down-regulate the expression of transcripts involved in connective tissue degradation and cytoskeleton re-organization (**j** & **k**). Values are log fold change (**d**–**g**) and actual fold change (**h**–**k**) ± SEM (*p < 0.05; **p < 0.01; ***p < 0.001 by un-paired *t* test)

We identified 61 and 124 DE genes in HS as well as 297 and 364 DE genes in FT groups at 1 week and 2 weeks, respectively (Fig. 1a, b). Comparisons of commonly deregulated genes over time revealed 39 and 58 DE genes in HS and FT groups, respectively (Fig. 1a–c). Comparisons between the two injury severities showed higher number of DE genes after FT compared to HS injury at all time-points (Fig. 1a–g). Pathway analysis identified *C4a* and *C4b* as the only transcripts associated with immune responses that were upregulated in HS group at 1 and 2 weeks post-injury (Fig. 1h). Simultaneously, at 2 weeks after HS, other genes linked to immune response and *Il13* signaling via JAK-STAT pathway were down-regulated including *Chitinase 3 L3 (Chi3l3)*, *Mmp8* and *Rsnb* (Additional file 3: Table S2). On the other hand, at 1 week after FT astrocytes displayed pronounced up-regulation of numerous transcripts associated with immune response, inflammation and natural killer cell cytotoxicity (Fig. 1h, i). At 2 weeks after FT, astrocytes displayed down-regulation of transcripts involved in proteolysis and connective tissue degradation, mitotic cell division and cytoskeleton remodeling (Fig. 1j, k).

Taken together, these results suggest that (1) the number of DE genes in astrocytes are higher after FT compared to HS injury, (2) astrocytic response after injury depends on both time- and lesion severity, (3) at 1 week after HS astrocytes only marginally participate in inflammatory process followed by inhibition of immune response at 2 weeks after HS and (4) at 1 week after FT astrocytes promote immune response and inflammation, followed by inhibition of connective tissue degradation and reduced proliferation.

### Resident mature astrocytes express neuronal progenitors and GABAergic markers associated with neural stem cell marker Fgfr4 over-expression after SCI

**Fig. 2:**
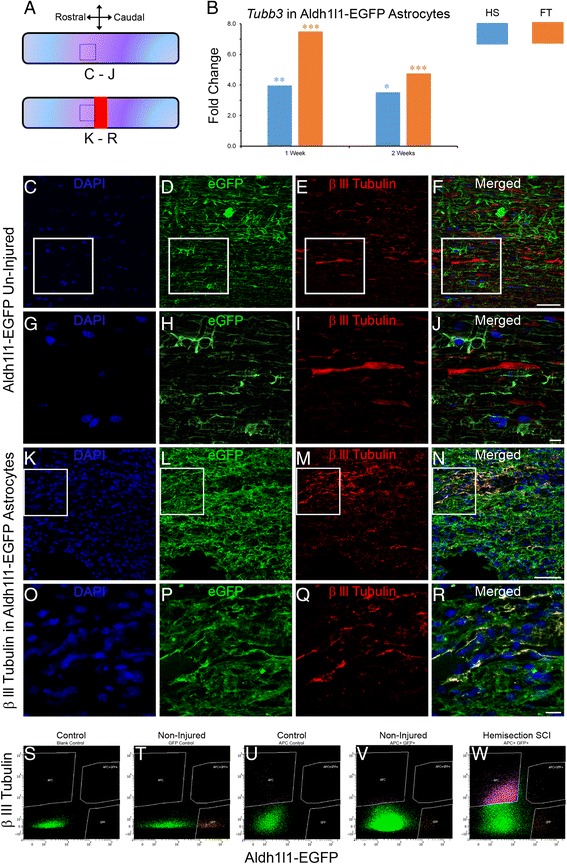
Astroglial conversion into neuronal progenitor cells after SCI. Schematic drawing of longitudinal spinal cord sections from non-injured control and after FT illustrating the lesion site (red rectangle) and reference frames for displayed fields of view (**a**). Bar graph displaying up-regulation of βIII-tubulin (*Tubb3*) transcript in astrocytes at different stages after SCI (**b**). Confocal micrographs of astrocytes in un-injured control showing no eGFP/βIII-tubulin co-expression (**c**–**j**). Confocal micrographs confirming βIII-tubulin protein expression in a sub-population of astrocytes in Aldh1l1-EGFP mice at 2 weeks after FT (**k**–**r**). Representative flow cytometry analysis dot plots displaying eGFP/βIII-tubulin co-expression only in injured Aldh1l1-EGFP spinal cord 2 weeks after HS (**s**–**w**). Surrounded areas, designed as “APC + GFP+”, correspond to the eGFP/βIII-tubulin co-expressing cells. The X and Y-axis represent fluorescent intensity for GFP and βIII-tubulin, respectively. **s**–**u** represent multiple controls necessary for the FACS (see material and methods for details). Scale bars (**c**–**f**, **k**–**n**): 50 μm and (**g**–**j**, **o**–**r**): 10 μm. **a** Values are actual fold change ± SEM (*p < 0.05; **p < 0.01; ***p < 0.001 by un-paired *t* test). **c**–**j** non-injured and (**k**–**r**) FT 2 weeks

**Fig. 3:**
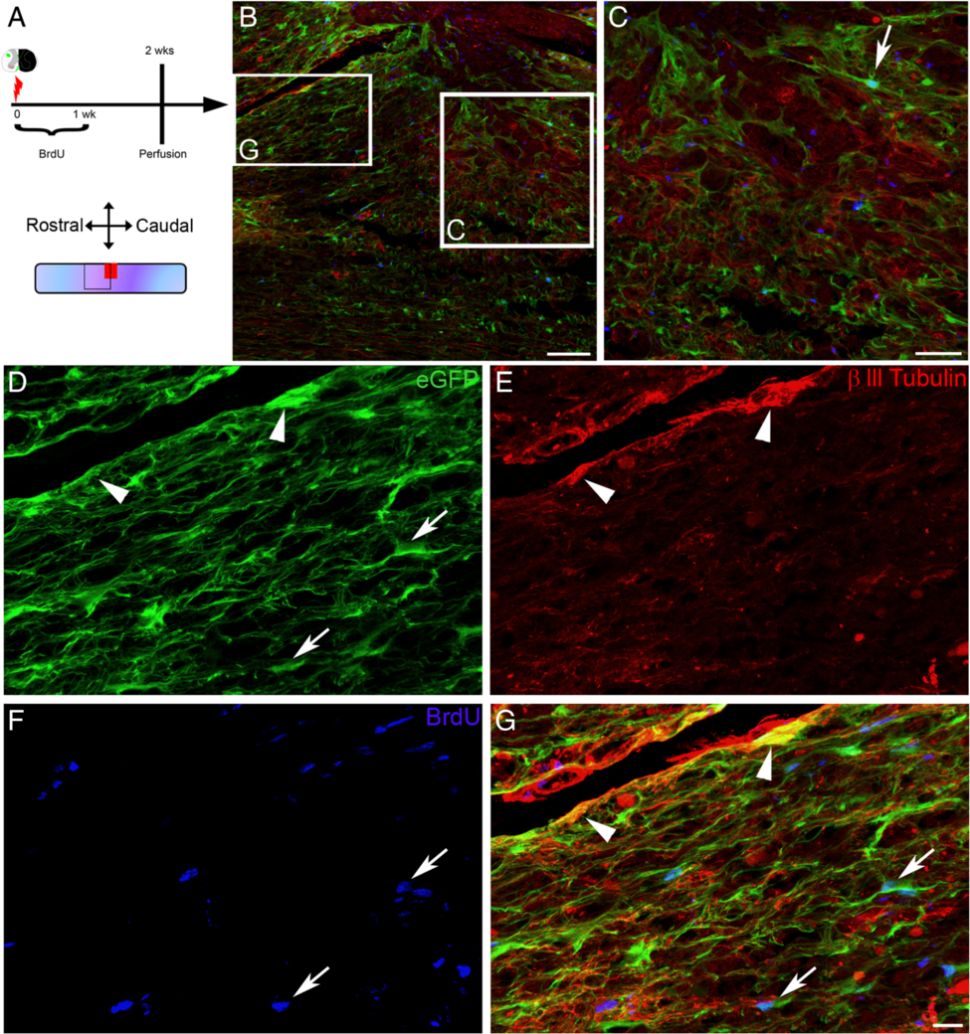
Transdifferentiation of local mature astrocytes into neuronal progenitors after SCI. Experimental time line for BrdU incorporation into newly formed astrocytes (**a**). Confocal mosaic micrograph showing the lesion site and reference frames for displayed fields of view in C and G (**b**). Confocal micrographs confirming BrdU incorporation into newly proliferated astroglia (arrows in **c**, **d**, **f** & **g**) but not in transdifferentiated astrocytes (arrowheads in **d**, **e** & **g**) in Aldh1l1-EGFP mice 2 weeks after HS. Scale bars (**b**): 100 μm, (**c**): 50 μm and (**d**–**g**): 20 μm. **b**–**g** HS 2 weeks

**Fig. 4:**
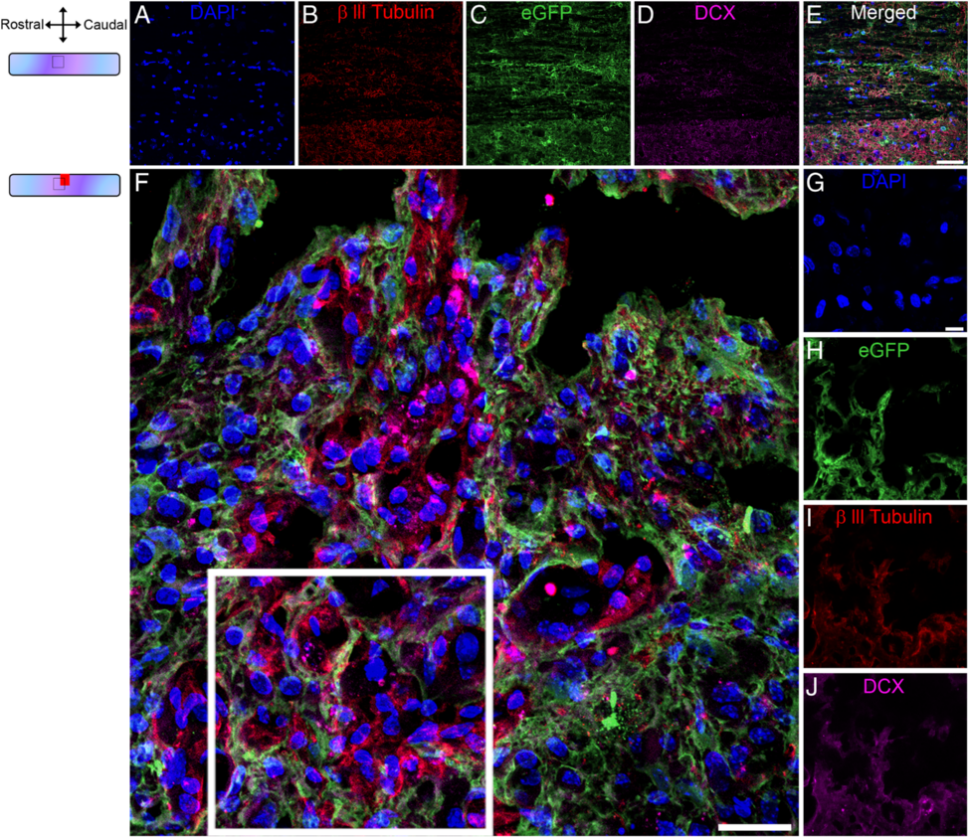
Doublecortin expression in astrocytes after SCI. Confocal micrographs showing DAPI (**a**), βIII-tubulin (**b**), astrocytes (eGFP, **c**) and DCX (**d**) expression in un-injured mice. No βIII-tubulin/eGFP/DCX co-expression was observed in un-injured astrocytes (**e**). Following SCI, transdifferentiated (eGFP/βIII-tubulin positive) astrocytes also expressed DCX (**f** & **h**–**j**). DAPI (**g**). Scale bars (**a**–**e**): 50 μm, (**f**): 25 μm, (**g**–**j**): 10 μm. **a**–**e** non-injured and (**f**–**j**) HS 2 weeks

Pathway analysis in astrocytes identified “neural stem cell lineage” at 2 weeks after FT (Additional file 3: Table S2) through the up-regulation of the neuronal progenitor gene βIII-tubulin (*Tubb3*, also known as *TUJ1*, Additional file 4: Figure S2). Increased *Tubb3* expression in astrocytes was observed at 1 and 2 weeks after HS and FT (Fig. 2b). Immunohistochemistry analysis confirmed βIII-tubulin expression in a sub-population of astrocytes located adjacent to micro-cavities within 750 μm distance to the lesion site (Fig. 2k–r). βIII-tubulin expression in astrocytes was also associated with a change in cell morphology from typical stellate shape to classical neuronal progenitor cells with bipolar or multipolar processes (Fig. 2o–r). Astrocytes in non-injured controls showed no βIII-tubulin expression (Fig. 2c–j). We then used FACS to deepen eGFP/βIII-tubulin co-expression analysis at single-cell level (Fig. 2s–w). Unlike non-injured controls, eGFP/βIII-tubulin co-expressing cells were specifically observed after HS (Fig. 2v & w). FACS analysis revealed that at 2 weeks after HS injury 14.96 % of astrocytes expressed βIII-tubulin (Fig. 2w). To examine the origin of eGFP/βIII-tubulin co-expressing cells, we then carried out BrdU injection in Aldh1l1-EGFP mice and found no BrdU incorporation in eGFP/βIII-tubulin co-expressing cells, suggesting that these cells are derived from resident mature astrocytes (Fig. 3a–g). To determine whether transdifferentiated astrocytes express other neuronal progenitor markers, we used doublecortin immunostaining (DCX, another classical neuronal progenitor marker) and found that eGFP/βIII-tubulin co-expressing cells also express DCX (Fig. 4f–j). Astrocytes in non-injured controls showed no βIII-tubulin or DCX expression (Fig. 4a–e).

**Fig. 5:**
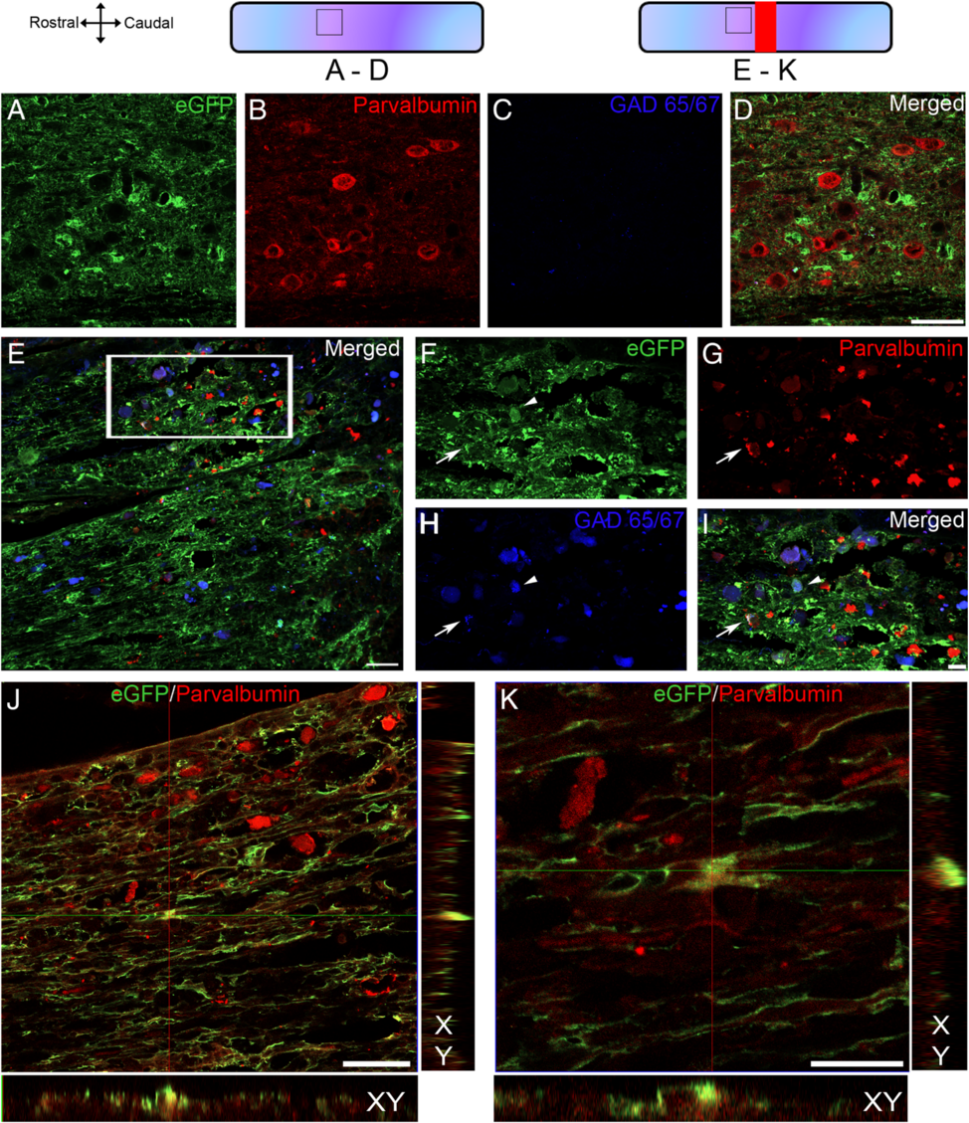
Astroglial transdifferentiation into GABAergic interneurons after SCI. Confocal micrographs showing parvalbumin and GAD 65/67 immunostaining in un-injured (**a**–**d**) and 6 weeks after FT lesion in Aldh1l1-EGFP mice (**e**–**k**). No eGFP/parvalbumin co-expression was observed in astrocytes of un-injured mice (**a**–**d**). Confocal mosaic micrograph showing the reference frames for fields of view displayed in F–I (**e**). At 6 weeks after FT triple eGFP/parvalbumin/GAD 65/67 expression was present within somata and primary cellular processes (arrow in **f**–**i**). A proportion of cells expressed double eGFP/GAD (arrowheads **f**–**i**) or eGFP/parvalbumin (**j** & **k**) proteins. Small insets in **j** and **k** are orthogonal projections of confocal z-stacks, which show eGFP co-localization with parvalbumin (confocal z-stack with XY, XZ, and YZ views). Scale bars(**a**–**d** & **e** & **j**) 50 μm and (**f**–**i** & **k**): 20 μm. (**a**–**d**) non-injured and (**e**–**k**) FT 6 weeks

**Fig. 6:**
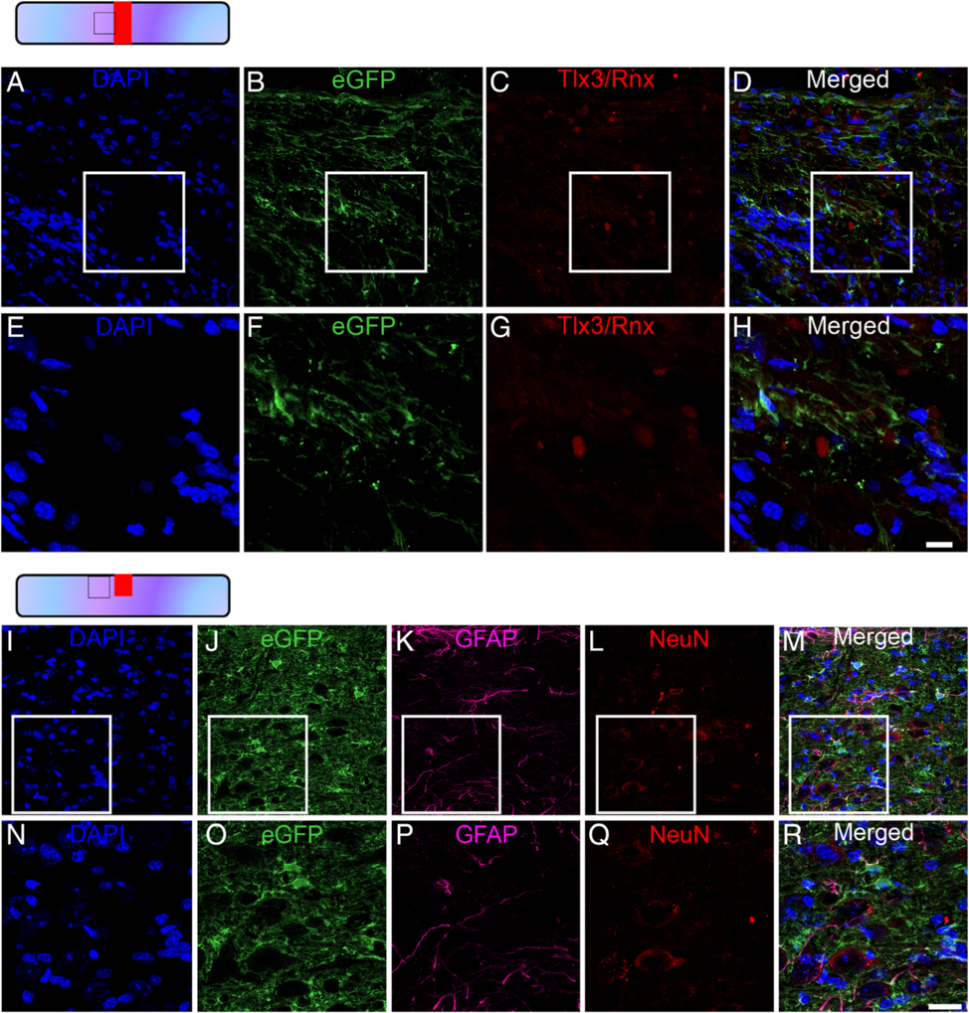
Lack of astroglial transdifferentiation into glutamatergic neurons after SCI. Confocal micrographs confirming no Tlx3/Rnx (**a**–**h**) and NeuN (**i**–**r**) co-expression by astroglial populations in Aldh1l1-EGFP mice after lesion. Scale bars (**a**–**d**, **i**–**m**): 50 μm, (**e**–**h**, **n**–**r**): 20 μm. **a**–**h** FT 2 weeks and (**i**–**r**) HS 6 weeks

To examine whether astrocytes-derived neuronal progenitors are subsequently converted into neurons, we performed immunohistochemistry to detect parvalbumin and glutamate decarboxylase (GAD 65/67) as markers for mature inhibitory GABAergic interneurons, as well as T-cell leukemia homeo box 3 (Tlx3/Rnx) and neuronal nucleus (NeuN) as glutamatergic and mature neuronal markers in Aldh1l1-EGFP mice (Figs. 5 and 6). In non-injured animals, astrocytes displayed typical stellate morphology with no parvalbumin or GAD 65/67 expression (Fig. 5a–d). Following lesion astrocytes displayed elongated morphology and showed parvalbumin expression within somata and primary processes (Fig. 5e–k). High magnification confocal analysis confirmed eGFP/parvalbumin co-expression in Aldh1L1-EGFP mice after lesion (Fig. 5j, k). The GABAergic interneuron identity of astrocytes-derived cells was further demonstrated by staining with antibody against GAD 65/67 (Fig. 5h, i). SCI-induced reactive astrocytes did not express glutamatergic or mature neuronal markers such as Tlx3/Rnx (Fig. 6a–h) and NeuN (Fig. 6i–r).

**Fig. 7:**
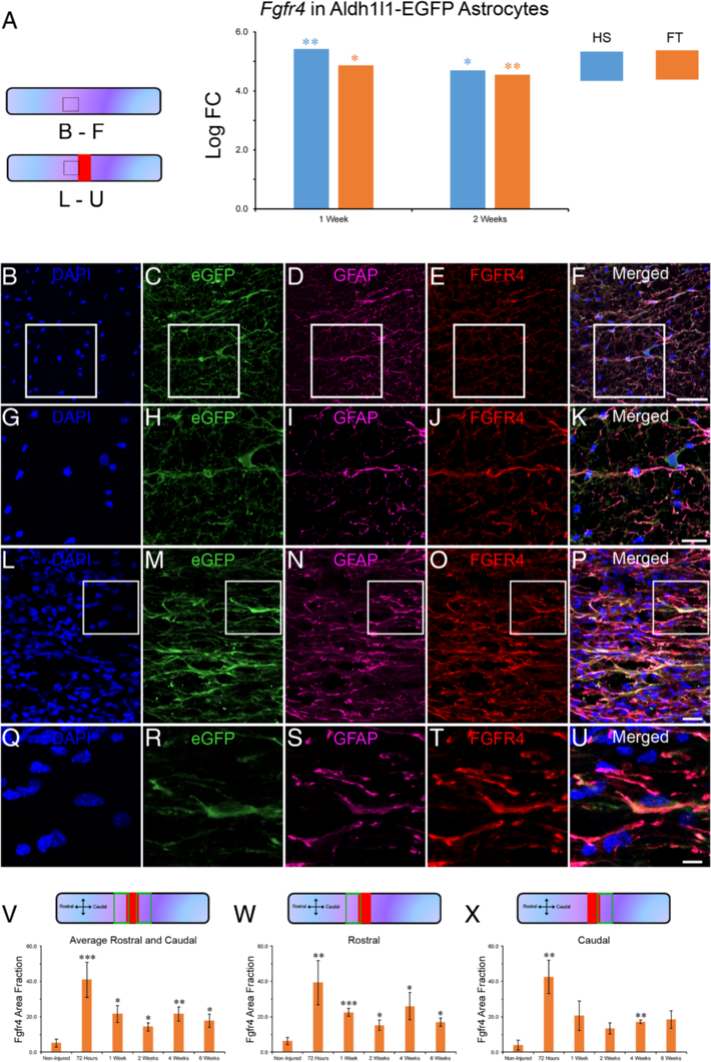
Up-regulation of *Fgfr4* in astrocytes after SCI. Bar graph displaying increased *Fgfr4* transcript expression in astrocytes (**a**). Confocal micrographs confirming astrocytic Fgfr4 protein over-expression after injury in Aldh1l1-EGFP mice (**l**–**u**) as compared to un-injured mice (**b**–**k**). Morphologically, Fgfr4 expression was identical to GFAP and eGFP profiles in Aldh1l1-EGFP mice. DAPI staining (**b**, **g**, **l** & **q**). Bar graphs displaying increased Fgfr4 protein expression at multiple stages after FT injury (**v**–**x**). Note that injury-induced increase in Fgfr4 protein expression is shown as an average of both rostral and caudal segments combined (green rectangles, **v**) as well as rostral (**w**) and caudal (**x**) segments individually. Scale bars (**b**–**f**): 50 μm, (**g**–**k**): 20 μm, (**l**–**p**): 25 μm, (**q**–**u**): 10 μm. **a** Values are actual fold change ± SEM (*p < 0.05; **p < 0.01 by *t* test). **v**–**x** Values are area fractions ± SEM (*p < 0.05; **p < 0.01; ***p < 0.001 by un-paired *t* test). **b**–**k** un-injured and (**l**–**u**) FT 4 weeks

To investigate the potential mechanisms responsible for astroglial transdifferentiation, we analyzed the expression of neurogenic markers in astrocytes after injury. We observed an increased expression of the gene encoding for fibroblast growth factor receptor 4 (*Fgfr4*), a neural stem cell marker [17], at 1 and 2 weeks after both lesion severities (Fig. 7a). Morphologically, Fgfr4 protein expression was evident in elongated astrocytic cytoskeleton and closely matched GFAP expressing profiles in non-injured control (Fig. 7b–k) and injured spinal cord (Fig. 7l–u). SCI triggered a clear increase in Fgfr4 expression in astrocytes located adjacent to the lesion site (Fig. 7o). Average Fgfr4 area fraction expression 1000 μm^2^ rostral and 1000 μm^2^ caudal to the lesion site revealed significant increase compared to non-injured throughout 6 weeks after FT injury (Fig. 7v). The peak in Fgfr4 protein expression was observed at 72 h with over 8-fold increase in area fraction compared to non-injured control (Fig. 7v). Further analysis revealed greater increase in Fgfr4 expression rostral compared to caudal segment of the lesion site (Fig. 7w, x).

Altogether, these data demonstrate SCI-induced astrocytic conversion towards neuronal progenitor and subsequently GABAergic interneurons lineage. Astrocytic transdifferentiation is strongly associated with over-expression of the neural stem cell marker Fgfr4.

### Glial transdifferentiation continues up to 6 weeks post-lesion

**Fig. 8:**
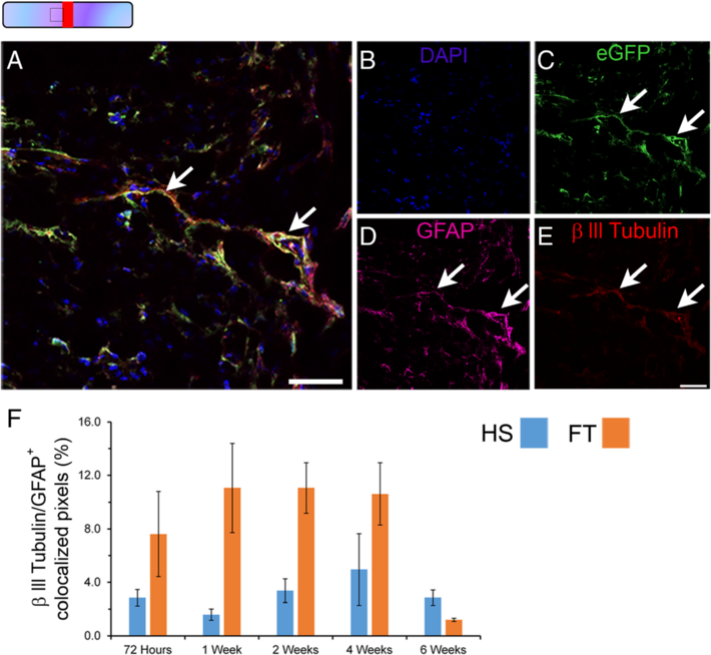
Astroglial transdifferentiation continues up to 6 weeks after different lesion severity. Confocal micrographs showing merged of DAPI (**b**), eGFP (**c**), GFAP (**d**) and βIII-tubulin (**e**) expression in Aldh1l1-EGFP mice 4 weeks after FT injury (**a**). Quantitative analysis of GFAP/βIII-tubulin (**f**) co-expressing cells (arrows) in the 2 lesion severities at multiple stages after SCI. Scale bars (**a**–**e**): 50 μm. Bars represent mean ± SEM. **a**–**e** FT 4 weeks

We finally examined SCI-induced astroglial transdifferentiation in adult spinal cord at multiple stages after different lesion severity (Fig. 8). Time-course analyses in Aldh1l1-EGFP mice revealed that astrocytic co-expression of the βIII-tubulin protein starts as early as 72 h following injury and showed a peak between 1 and 2 weeks post-lesion reaching over 11.1 % after FT injury (Fig. 8f). By 6 weeks after lesion only 2.9 and 1.2 % of astrocytes continued to express βIII-tubulin in HS and FT groups, respectively (Fig. 8f).

These data demonstrate that astrocyte transdifferentiation towards neuronal lineage starts early after injury peaking by 1 week post-lesion and continues to a lower level up to 6 weeks (the longest time-point we have investigated in this study) following both injury severities.

## Discussion

**Fig. 9:**
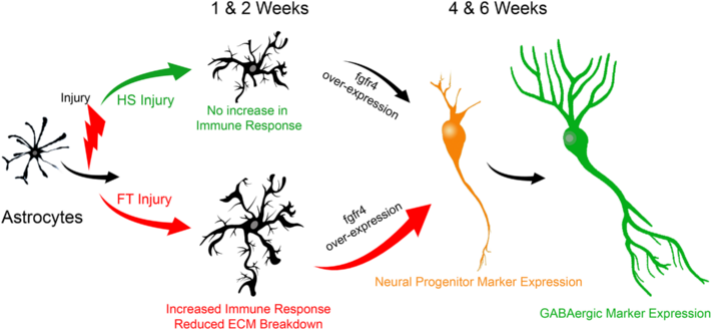
Schematic cartoon illustrating the overall astrocytic response after SCI. Astrogliosis after SCI depends on both time post-injury and lesion severity. At 1 week after HS astrocytes undergo moderate activation and do not promote an immune response by 2 weeks after HS SCI. On the other hand, at 1 week following FT astrocytes undergo marked activation and actively promote immune response/inflammation followed by reduced extracellular membrane breakdown at 2 weeks after FT. Concurrently, a sub-population of local astrocytes transdifferentiates into neuronal progenitor and subsequently GABAergic interneurons via fgfr4 over-expression

Findings of the current study indicate that astrocytes undergo specific transcriptomic alterations after SCI that depends on both time post-lesion and injury severity. Importantly, SCI induces astrocytic conversion (both morphologically and via βIII-tubulin and DCX expressions) towards neuronal progenitors and subsequently GABAergic interneurons (see Fig. 9). Increased astroglial transdifferentiation is associated with over-expression of the neural stem cell marker Fgfr4 and continues up to 6 weeks after CNS injury. These findings represent the first cell-specific transcriptomic analysis of astrocytes after different lesion severities providing a novel insight into astroglial plasticity.

### Heterogeneous astrocytic response after SCI

Astrogliosis is a pathological hallmark of SCI involving not only morphological hypertrophy but also pronounced changes at molecular and functional levels [2, 18]. Contradictory reports on astrocytic response after SCI has been reported including increased [3] and inhibition of neuroinflammation [4]. Our data provide the first evidence of a heterogeneous astrocytic response after SCI that depends on both time post-lesion and injury severity. Specifically, astrogliosis after FT may be detrimental for regeneration by promoting neuroinflammation (1 week) and inhibiting extracellular re-modeling (2 weeks), whereas astrocytic response after HS may be beneficial by an absence of increase (1 week) and down-regulation of immune response (2 weeks). Our data provide the first evidence for the general consensus that astrocytic response after SCI depends on numerous factors including lesion severity, time post-injury as well as distance to the lesion epicenter [2, 18]. Heterogeneous astrocytic response has been reported after lipopolysaccharide injection and stroke [14] as well as at different stages of Alzheimer’s disease [13]. Altogether, these findings demonstrate that astrogliosis depends on multiple factors including the type of inducing stimulus, time after activation and injury severity.

### Transdifferentiation of astrocytes towards neuronal lineage after SCI

Another important finding from our study is the autologous transdifferentiation of resident mature astrocytes into neuronal progenitors and subsequently GABAergic interneurons after SCI. Previous studies have reported SCI-induced expression of progenitor markers by astrocytes including Sox2 and nestin [19, 20]. However, contrary to our findings, no study had shown spontaneous astroglial conversion into neuronal progenitors or adult neurons. One reason for this discrepancy may be due to markers used to label astrocytes. All the mentioned studies had relied on GFAP as a marker of astrocytes, whereas we have used Aldh1L1 expression that has been shown to be a more pan astrocytic marker than GFAP [15]. Our findings provide evidence that a small population of resident astrocytes are spontaneously reprogrammed towards GABAergic interneurons in vivo after CNS injury. Lack of BrdU incorporation into eGFP/βIII-tubulin co-expressing cells confirmed that the origin of these cells are transdifferentiated resident astrocytes rather than newly formed astrocytes. Comparison between the two lesion severity showed greater level of eGFP/βIII-tubulin co-expression after severe SCI, suggesting that greater astrocytic activation and enlarged lesion area may also play important roles in astrocyte transdifferentiation toward neuronal lineage.

More recent findings suggest that enforced expression of neurogenic factors including *Notch* [21], *Sox2* [22], *NeuroD1* [23], *Shh* [24], *Ascl1*, *Brn2* and *Myt1l* [25] can directly convert astrocytes into functional neurons in vivo. However, we found no significant over expression of these transcripts, suggesting that other signaling molecules may be involved in autologous astrocytic transdifferentiation towards neuronal lineage after SCI. In this regard, we have identified the fibroblast growth factor receptor 4 (*Fgfr4*) as a potential autologous modulator of astrocytic transdifferentiation following SCI. This is, to our knowledge, the first demonstration that Fgfr4 protein is expressed in astroglial cells and its expression is increased in activated astrocytes following CNS injury. Interestingly, the peak increase in Fgfr4 protein expression in astrocytes was observed at 72 h post-lesion, whilst the peak increase in βIII-tubulin protein expression was not observed till 1 week post-lesion, suggesting that Fgfr4 protein over-expression precedes astroglial conversion towards neuronal lineage.

The 4 subfamilies of fibroblast growth factor receptors (Fgfr1-4) play multiple functions in progenitor cell proliferation and neuronal differentiation [26]. Endogenously expressed Fgfr4 ligand (Fgf4) promotes embryonic stem cell differentiation towards neuronal lineage [27] and elevated Fgf4 is essential for astrocytic conversion into neuronal progenitor-like cells [28]. We found no changes in *Fgfr1* and *Fgfr2* transcripts, whilst the gene encoding for *Fgfr3* was actually down-regulated 1 week after HS injury. Taken together, these findings suggest that Fgf4 action on astrocytes is most likely due to the activation of Fgfr4 rather than other fgf receptor family members. Elevated Fgfr4 expression in astrocytes may thus be critical for their transdifferentiation into neuronal progenitor-like cells after SCI.

A major difference between our findings and published reports is that whereas others had introduced extrinsic neurogenic factors to force astrocyte transformation into neurons, we observed here a spontaneous phenomenon. Equally important, none of the previous studies had shown time-course analysis of glial transdifferentiation in vivo. Altogether, these findings suggest that optimizing autologous glial transdifferentiation may be more effective in replacing neuronal loss and improving functional recovery following injury. The functional effect of astroglial transformation into neurons awaits further studies.

## Conclusion

Our data highlight that astrocytic response after injury is both time -and severity-dependent involving inhibition and stimulation of immune response after moderate and severe SCI, respectively. Importantly, we show that SCI induces astrocytic conversion towards GABAergic interneurons via transition through neural stem cell (Fgfr4) and neuronal progenitors (βIII-tubulin/DCX) stages. Further studies are required to establish whether astrocyte-derived interneurons can form functional synapses with the neighboring neurons and facilitate neurotransmission across the lesion site. Enforced Fgfr4 and βIII-tubulin expression in reactive astrocytes may promote greater astroglial transdifferentiation towards neuronal lineage and provide a novel therapeutic approach to replace neuronal demise and promote functional recovery after CNS injury.

## Additional files

Additional file 1: Figure S1. Specific eGFP expression of astroglial cells in Aldh1l1-EGFP mice. Confocal micrographs showing astrocytic eGFP expression in Aldh1l1-EGFP mice that was confirmed using GFAP immunostaining (A–C). Fluorescent micrographs showing lack of eGFP expression in microglia/macrophages (Iba1, D&E), oligodendrocytes (MBP, F) and neuronal (MAP2, G) populations in Aldh1l1-EGFP mice. Scale bars (A, F&G): 50 μm, (B&C): 10 μm, (D): 30 μm, (E): 15 μm. Representative flow cytometry analysis dot plot displaying control (H) and eGFP-expressing astrocytic profiles from un-injured (I) as well as after injury (J). Surrounded areas, designed as “P4”, correspond to the eGFP-expressing astrocytes. The X-axis represents fluorescent intensity and the Y axis cell size. RNA quality isolated from FACSed astrocytes (K). (TIF 20779 kb)
Additional file 2: Table S1. Database of differential expression comparison of activated astrocyte RNA-Seq data relative to non-injured control astrocyte at 1 and 2 weeks after hemisection and full transection injuries. (XLSX 90 kb)
Additional file 3: Table S2. Pathway analysis of differentially expressed genes in activated astrocytes after hemisection and full transection injuries. (XLSX 19 kb)
Additional file 4: Figure S2. Induction of transdifferentiation pathways in astrocytes after SCI. Gene ontology pathway map analysis of deregulated genes in astrocytes demonstrate the induction of a neural development pathway. Thermometers indicate the deregulation of the gene (red: up-regulated; blue: down-regulated). Thermometers with “1” represent gene deregulation in astrocytes. Interactions between objects: green (positive or activation); red (negative or inhibition); grey (unspecified); B: Binding (physical interaction between molecules). (TIF 3235 kb)

## Abbreviations

7-AAD: 7-aminoactinomycin
Aldh1L1: Aldehyde dehydrogenase 1 family member L1
DCX: Doublecortin
eGFP: Enhanced green fluorescent protein
FACS: Fluorescence-activated cell sorting
Fgfr4: Fibroblast growth factor receptor 4
FT: Full transection
GAD 65/67: Glutamate decarboxylase
HS: Hemisection
MMRRC: Mutant Mouse Regional Resource Centre
NeuN: Neuronal nucleus
NI: Non-injured
PBS: Phosphate base saline
PFA: Paraformaldehyde
RNA-Seq: RNA sequencing
SCI: Spinal cord injury
Tlx3/Rnx: T-cell leukemia homeo box 3
