# Supplementary figures and images for: Spinal cord injury induces astroglial conversion towards neuronal lineage
Source: Mol Neurodegener. 2016 Oct 6;11:68. doi: 10.1186/s13024-016-0133-0 (PMC5052929; doi:10.1186/s13024-016-0133-0)

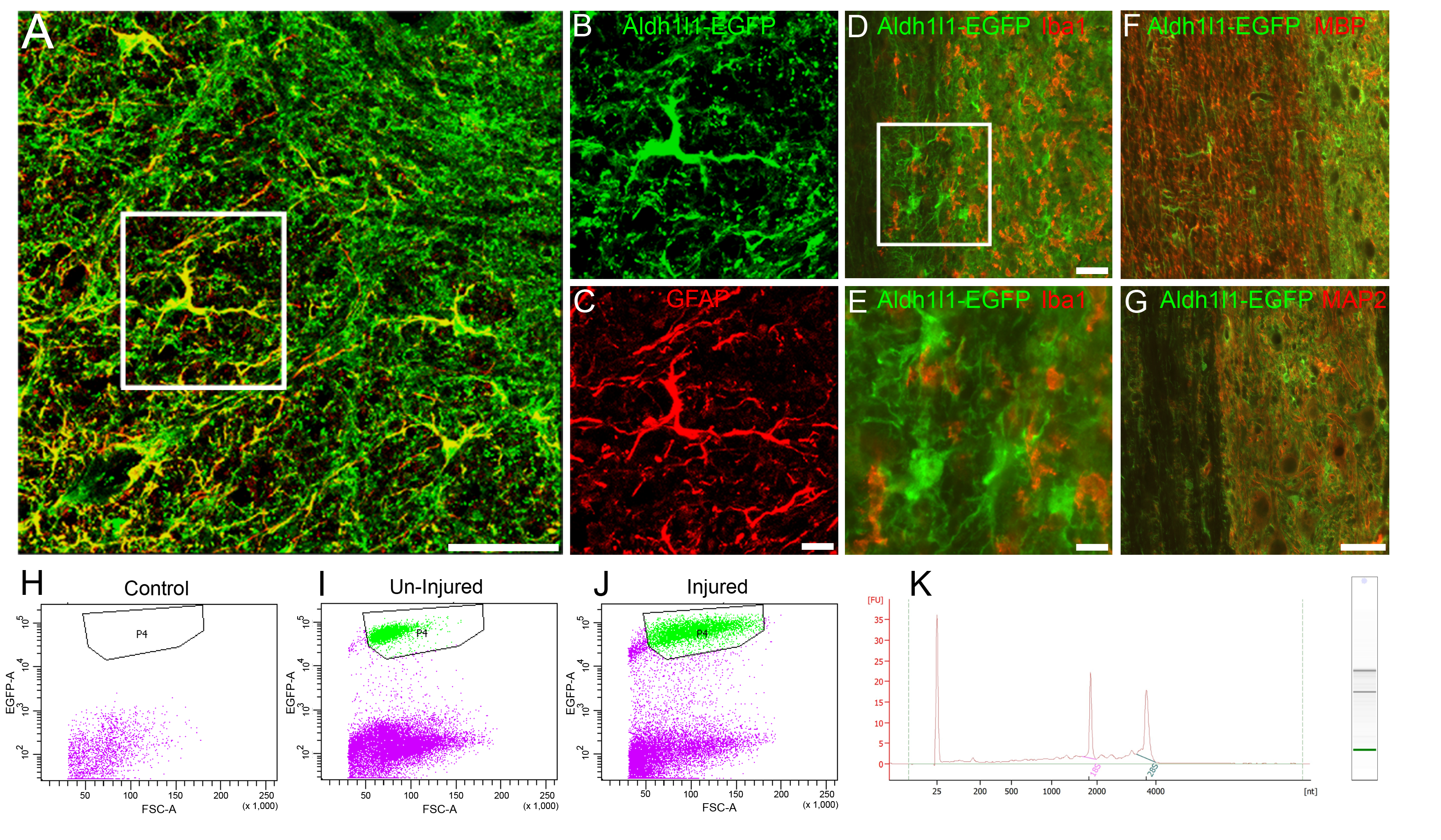

Supplement: Additional file 1: Figure S1. — Specific eGFP expression of astroglial cells in Aldh1l1-EGFP mice. Confocal micrographs showing astrocytic eGFP expression in Aldh1l1-EGFP mice that was confirmed using GFAP immunostaining (A–C). Fluorescent micrographs showing lack of eGFP expression in microglia/macrophages (Iba1, D&E), oligodendrocytes (MBP, F) and neuronal (MAP2, G) populations in Aldh1l1-EGFP mice. Scale bars (A, F&G): 50 μm, (B&C): 10 μm, (D): 30 μm, (E): 15 μm. Representative flow cytometry analysis dot plot displaying control (H) and eGFP-expressing astrocytic profiles from un-injured (I) as well as after injury (J). Surrounded areas, designed as “P4”, correspond to the eGFP-expressing astrocytes. The X-axis represents fluorescent intensity and the Y axis cell size. RNA quality isolated from FACSed astrocytes (K). (TIF 20779 kb) [file 13024_2016_133_MOESM1_ESM.tif]

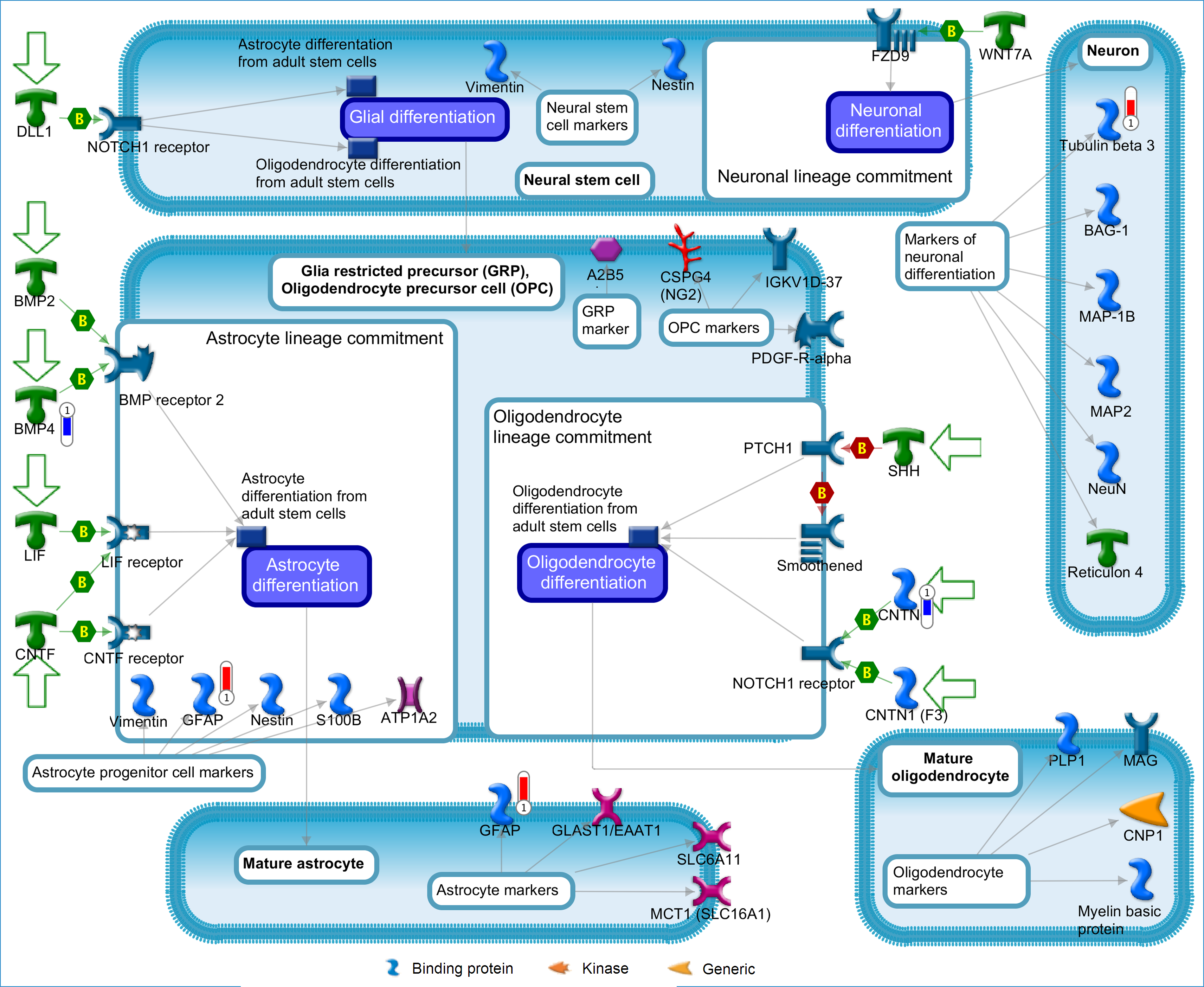

Supplement: Additional file 4: Figure S2. — Induction of transdifferentiation pathways in astrocytes after SCI. Gene ontology pathway map analysis of deregulated genes in astrocytes demonstrate the induction of a neural development pathway. Thermometers indicate the deregulation of the gene (red: up-regulated; blue: down-regulated). Thermometers with “1” represent gene deregulation in astrocytes. Interactions between objects: green (positive or activation); red (negative or inhibition); grey (unspecified); B: Binding (physical interaction between molecules). (TIF 3235 kb) [file 13024_2016_133_MOESM4_ESM.tif]
